# Can P-glycoprotein and β-tubulin polymorphisms be used as genetic markers of resistance in *Dirofilaria immitis* from Rio de Janeiro, Brazil?

**DOI:** 10.1186/s13104-018-3259-z

**Published:** 2018-02-23

**Authors:** Liliane Maria Valentim Willi, Norma Vollmer Labarthe, Luiz Ney d’Escoffier, Jonimar Pereira Paiva, Marcia Gonçalves Nobre de Miranda, Flavya Mendes-de-Almeida, Tânia Zaverucha do Valle

**Affiliations:** 1 Programa de Pós-Graduação em Medicina Veterinária – Clínica e Reprodução Animal, Faculdade de Veterinária, Universidade Federal Fluminense, Rua Vital Brazil Filho 64, Niterói, RJ 24230-340 Brazil; 2 Fundação Oswaldo Cruz, Av. Brasil 4365, Rio de Janeiro, RJ 21040-360 Brazil; 3 Laboratório de Imunomodulação e Protozoologia, Instituto Oswaldo Cruz, Fiocruz, Av. Brasil 4365, Rio de Janeiro, RJ 21040-360 Brazil; 4 Departamento de Medicina e Cirurgia Veterinária, Instituto de Veterinária, Universidade Federal Rural do Rio de Janeiro, BR-465, Km 7, Seropédica, RJ 23890-000 Brazil; 5 Médica Veterinária, Vet Ypiranga, Rua Ypiranga 107, Laranjeiras, Rio de Janeiro, RJ 22231-120 Brazil

**Keywords:** *Dirofilaria immitis*, Macrocyclic lactones, Genetic marker of resistance

## Abstract

**Objective:**

*Dirofilaria immitis*, the causative agent of canine heartworm infection, is worldwide the most important filarid to affect domestic dogs. Prevention of this infection is done by macrocyclic lactones, but some reports on the lack of efficacy have been published. Although the actual cause of resistance is unknown, single nucleotide polymorphisms (SNPs) on a P-glycoprotein ABC transporter and β-tubulin genes have been pointed out as candidates for genetic markers of resistance. We conducted a survey to verify the presence of these suggested genetic markers in microfilariae from 30 naturally infected dogs under macrocyclic lactones treatment living in an endemic area in the state of Rio de Janeiro.

**Results:**

The analysis of these specific SNPs demonstrated no sign of polymorphism on the P-glycoprotein loci, while 72 and 48% of the samples were polymorphic to the first and second SNPs on β-tubulin loci, respectively. This work demonstrates that the P-glycoprotein position 11 and 618 were not polymorphic and, therefore, not suitable as a genetic marker of resistance in Rio de Janeiro whereas both β-tubulin loci were polimorphic. This work points out the difficulty of finding a universal genetic marker for resistance.

**Electronic supplementary material:**

The online version of this article (10.1186/s13104-018-3259-z) contains supplementary material, which is available to authorized users.

## Introduction

The filarial nematode, *Dirofilaria immitis*, the causative agent of canine heartworm infection, is worldwide the most important filarid to affect domestic dogs. Since the 80’s, prevention of this infection is done by macrocyclic lactones (ML), but some reports on the lack of efficacy have been published in the last 12 years. Resistance to macrocyclic lactones (ML) is a major problem in small ruminants, horses and cattle, leading to a discussion on the way these drugs should be used in livestock farms [1]. In dogs, the first report on ML loss of efficacy in *D. immitis* prevention has been published in 2005 [2]. Resistance in canine worms should be more difficult to develop than in ruminant helminths [3] because stray and feral dogs, as well as wild canids, carry a population of parasites that is not exposed to drug selection and therefore represent a wide refugia. The refugia bring drug-susceptibility alleles that dilute out the resistant ones in the population, reducing the selection pressure for drug resistance [4]. Nevertheless, a study using microfilariae obtained from dogs from US and Japan has shown the genetically variability of *D. immitis,* suggesting that the development of resistance could be possible under a high selective pressure [5]. Studies conducted using microfilariae obtained from three dogs originally from Louisiana and Arkansas, USA, that were under unsuccessful ML heartworm preventive treatment demonstrated an excess of homozygosity, which might be a sign of selection [6]. In addition, these microfilariae presented high frequency of a GG–GG genotype on two single nucleotide polymorphisms (SNPs) at positions 11 and 618 of a P-glycoprotein (*Pgp*) ABC transporter gene (i.e. an homozygous G was present on both positions). These findings suggest that the *Pgp* genotype might be useful as a genetic marker to assay for low responding *D. immitis* in the field [7]. A total of 9 ABC transporter genes have been identified in *D. immitis* and both SNPs are located in the Dim-pgp-11 [8]. Interestingly, in *Parascaris equorum*, resistance to ML was correlated with the presence of 3 individual SNPs and with an increase in the level of Peq-pgp-11 mRNA [9].

β-tubulin (Tub) has also been pointed out as a candidate gene for carrying genetic markers for resistance since it presents two SNPs that are in linkage disequilibrium in resistant microfilariae [5].

In the present study microfilaremic dogs living in the state of Rio de Janeiro, Brazil, received ML monthly for 4 months and a month after the forth dose microfilariae were collected. We aimed to identify the presence of the genotype for the two SNPs in *the Pgp* and the two others in *Tub* genes in worms from the studied area.

## Main text

The study was conducted in two coastal areas of the state of Rio de Janeiro in sandbanks of the municipalities of Arraial do Cabo and Araruama, where the dogs are exposed to a high challenge, 80.8 and 58.4% respectively [10]. Both areas are located at sea level, apart from one another for 10 km. Each area is located in opposite sides of a salt water lagoon. Yearound climate conditions in the area favor mosquitoes population and therefore heartworm transmission may occur at any time of the year. Microfilaremic and antigenemic dogs were included in the study after positive SNAP 4DX and modified Knott’s [11] tests, once their microfilaraemia was over 300 microfilariae/mL of blood [12]. Dogs’ history of ML treatment was not taken into consideration as there was no trustful record of it. This study was approved by Fundação Oswaldo Cruz (FIOCRUZ) Institutional Animal Care and Use Committee (CEUA—LW-33/11). All dogs received four consecutive monthly doses of avermectin [ivermectin–0.06–0.12 mg/kg (Endogard, Virbac) or selamectin–6 mg/kg (Revolution, Zoetis)] following manufacturer’s guidelines. All doses were administered by the study team. Prior to treatment and a month after the last dose, microfilaremia was blind counted using 2 fixed giemsa-stained blood slides of each dog, each one counted by the same two researchers [12]. Each dog’s microfilaraemia was calculated by the mean of the 4 counts. Statistical tests were performed in GraphPad Prism software, using Wilcoxon test. A month after the last dose, microfilariae were isolated from 1 mL of fresh blood samples by filtration through polycarbonate membranes [13]. Microfilariae were fixed in isopropanol and kept under refrigeration. DNA from pools of 50–100 microfilariae from each dog was extracted using phenol/chloroform method [14] and submitted to PCR amplification with Platinum Taq DNA Polymerase (Invitrogen). The *Pgp* gene fragment (GenBank HM596853) and the first *Tub* fragment (position 28 to 719) were amplified using primers described elsewhere [5], while the second *Tub* fragment (position 2364–2961) was amplified using the primers 5′ TGCTGAGCTCACTCAACAGG 3′ and 5′ GAATGTTGCGCTCATCTTCA 3′. After confirmation by electrophoresis on a 1.5% agarose gel, the amplified fragments were sequenced using a 3730XL DNA analyzer system at the Sequencing Plataform at FIOCRUZ (Plataforma de Sequenciamento de DNA, PDTIS/FIOCRUZ). After sequencing, each individual chromatogram was analyzed in SeqScape 2.1 (Applied Biosystems) and sequences were aligned in BioEdit Sequence Alignment Editor 7.1.11 [15]. Sequences with multiple peaks along the whole fragment at least in two sequencings of the same pool were discarded.

**Fig. 1:**
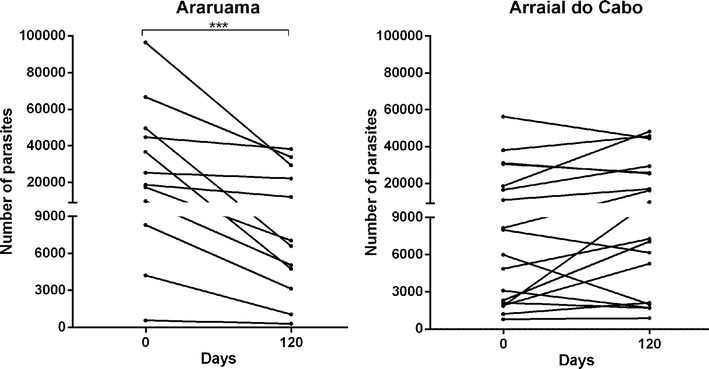
Microfilaria counting—Number of microfilariae/ml calculated from counting blood smears prior to treatment (0 days) and 30 days after treatment end (120 days). Treatment was performed with four consecutive monthly doses of avermectins (ivermectin-0.06–0.12 mg/kg or selamectin—6 mg/kg) following manufacturer’s guidelines (***P < 0.001, Wilcoxon test)

Counting of microfilariae before the first dose and 30 days after the last ML treatment dose showed that, in spite of the four doses, all 30 dogs remained microfilaremic. Microfilaraemia in Araruama canine samples declined (12/12; 100%, P = 0.0005), while in Arraial do Cabo canine samples microfilariae counting remained similar (7/18 declined; 39%, P = 0.2121). Ten dogs from Arraial do Cabo had an increase in microfilaremia, in spite of ML treatment (Fig. 1). Samples of microfilariae from all these dogs, 30 days after the end of treatment, were sequenced. Positions 11 and 618 of the *Pgp* fragment could be analyzed in 28 and 26 pool samples respectively, while position 561 and 2755 of the *Tub* gene could be analyzed in 25 and 27 pool samples respectively.

The analysis of *Tub* chromatograms (Additional file 1) showed the presence of heterozygosity on 72% of samples at position 561 (18/25) and 48% of samples at position 2755 (13/27), showing that these positions are polymorphic in *D. immitis* circulating among dogs in Rio de Janeiro. On the other hand, none of the *Pgp* chromatograms showed any sign of polymorphism as a single ‘A’ peak on position 11 and a single ‘G’ peak on position 618 were detected (Table 1). In spite of the SNPs found in the *Tub* gene, no relation was observed between increase in microfilaremia and presence of SNP (Fisher’s exact test, P = 0.1581) and SNPs could be found in dogs from both areas.

**Table 1:** Quantitative SNP results

Gene	Position	Microfilaria samples		
		Total number of samples	Number of samples with good readings	Number of samples with SNP
P-glycoprotein	11	30	28	Not found
	618	30	26	Not found
β-tubulin	561	30	25	18
	2755	30	27	13

Number of DNA samples from microfilaria pools of *Dirofilaria immitis* from the state of Rio de Janeiro analyzed for the presence of point polymorphisms in *P*-*glycoprotein* and -*tubulin* genes

According to our results, it seems that the described *Pgp* SNPs do not occur in worms of the studied area and therefore are not suited as genetic markers of worm resistance to ML in this area. The Tub SNPs, although present, did not seem to be related to resistance. This information raises the issue that their use might be restricted to a specific geographic region and could not be accepted globally. However, since the limit of detection for alternative alleles with Sanger sequencing is 15–20% [3] it must be considered that an undetectable low frequency of SNPs could be present in these microfilariae as we used pools.

Nevertheless the comparison of the adult worms from US and Japan dogs showed important differences among the polymorphic loci [5]. This fact alone highlights the difficulty in finding a global genetic marker. Likewise in other helminths, such as *Fasciola hepatica*, it has been demonstrated that the SNP T687G of the *Pgp* gene is a good genetic marker for drug resistance in some regions of the globe but not in others [16].

On the other hand, the GG–GG genotype described by Bourguinat et al. is not expected to be the actual cause of the resistance, but rather a surrogate marker for resistance [7] and therefore it may be absent in some resistant individuals. Indeed, the sequencing of the *Pgp* locus in the *D. immitis* MP3 strain, which is considered to have at least a proportion of resistant individuals, did not reveal the GG–GG genotype neither [7]. Nevertheless, the absence of polymorphism in these *Pgp* loci by itself argues against the use of these SNPs as genetic markers in *D. immitis* of the studied area.

It must also be noted that 10 dogs from the municipality of Arraial do Cabo increased the number of microfilariae despite the use of avermectin, whereas none from Araruama presented the same increase. However, the presence of SNPs in the studied loci do not account for that difference. Since 8 years before, in that exact area of Arraial do Cabo [17] microfilaremic dogs received 3 cycles of doxycycline at 6-months intervals, it may be inferred that the antibiotic could have impacted the heartworm *Wolbachia* population in the area. At the former study the first treatment cycle caused a sharp drop in microfilaraemia. After 6 months microfilaraemia rebounded and the other 2 cycles did not reduce microfilaraemia as much as the first did. Since *Wolbachia* is known to guarantee worm vital functions [18], to colonize the worm’s hipodermis, to resist to doxycycline although in low numbers [19] and to guarantee the development of infective larvae to the adult stage [20], it may be hypothesized that doxycycline surviving *Wolbachia* interfere with heartworm ML uptake and, therefore, turn the worms less susceptible to the effects of ML.

## Limitations

Analysis was focused in pre-described SNPs, as stated in the main text. We have not looked for other SNPs because our interest was to search for the same polymorphisms described in Lousiana, US. This objective was accomplished but we cannot exclude the presence of SNPs in other positions that would still put *Pgp* as a candidate gene for bearing a genetic or even functional marker of resistance. At the same time, sequencing was carried out using pools of microfilaria. As Sanger sequencing have a limit of detection of 15–20%, there is a change that we could not visualize an existing SNP if it was present in less than 20% of microfilariaes from a pool. Working in pools was a methodological choice. We preferred to enlarge our data in the number of dogs examined, instead of analysing individual microfilaria in only two or three dogs.

## Additional file

**Additional file 1.** Sequencing data. Chromatograms obtained by sequencing the samples of microfilariae pools of *Dirofilaria immitis*. Each figure shows all chromatograms for a given position of either *Pgp* or *Tub* gene.

## Abbreviations

ML: macrocyclic lactone
SNP: single nucleotide polymorphism
PGP: P-glycoprotein
TUB: β-tubulin
PCR: polymerase chain reaction
