# Supplementary material for: Can P-glycoprotein and β-tubulin polymorphisms be used as genetic markers of resistance in Dirofilaria immitis from Rio de Janeiro, Brazil?
Source: BMC Res Notes. 2018 Feb 23;11:152. doi: 10.1186/s13104-018-3259-z (PMC5824453; doi:10.1186/s13104-018-3259-z)
Supplement: Supplementary file 1 — Additional file 1. Sequencing data. Chromatograms obtained by sequencing the samples of microfilariae pools of Dirofilaria immitis. Each figure shows all chromatograms for a given position of either Pgp or Tub gene. [file 13104_2018_3259_MOESM1_ESM.pdf]

Sequencing data

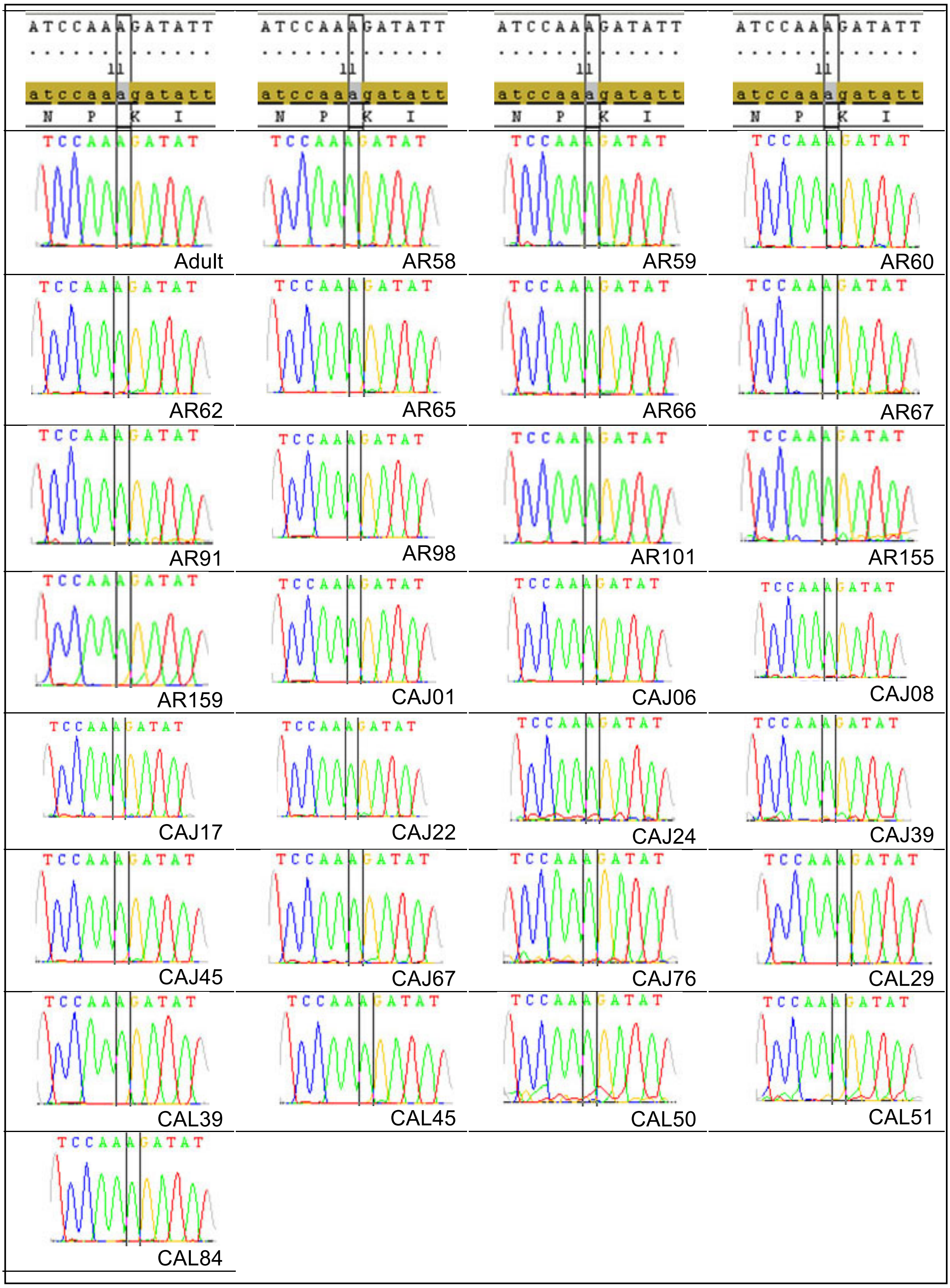

Figure 01: Chromatograms obtained by sequencing the samples of microfilariae pools of *Dirofilaria immitis* sequenced for partial gene P-glycoprotein with marking position 11.

Sequencing data

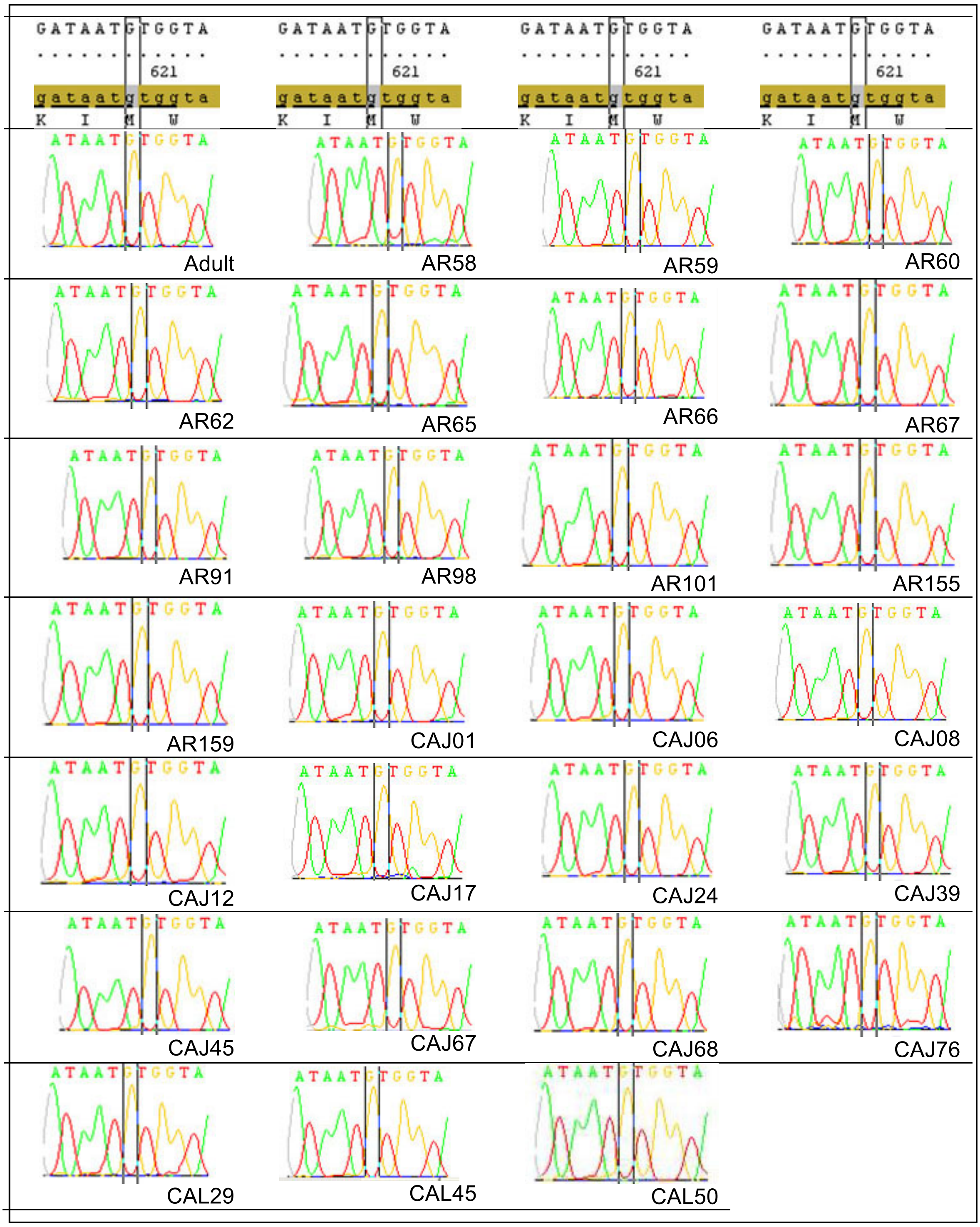

Figure 02: Chromatograms obtained by sequencing the samples of microfilariae pools of *Dirofilaria immitis* sequenced for partial gene P-glycoprotein with marking position 618.

Sequencing data

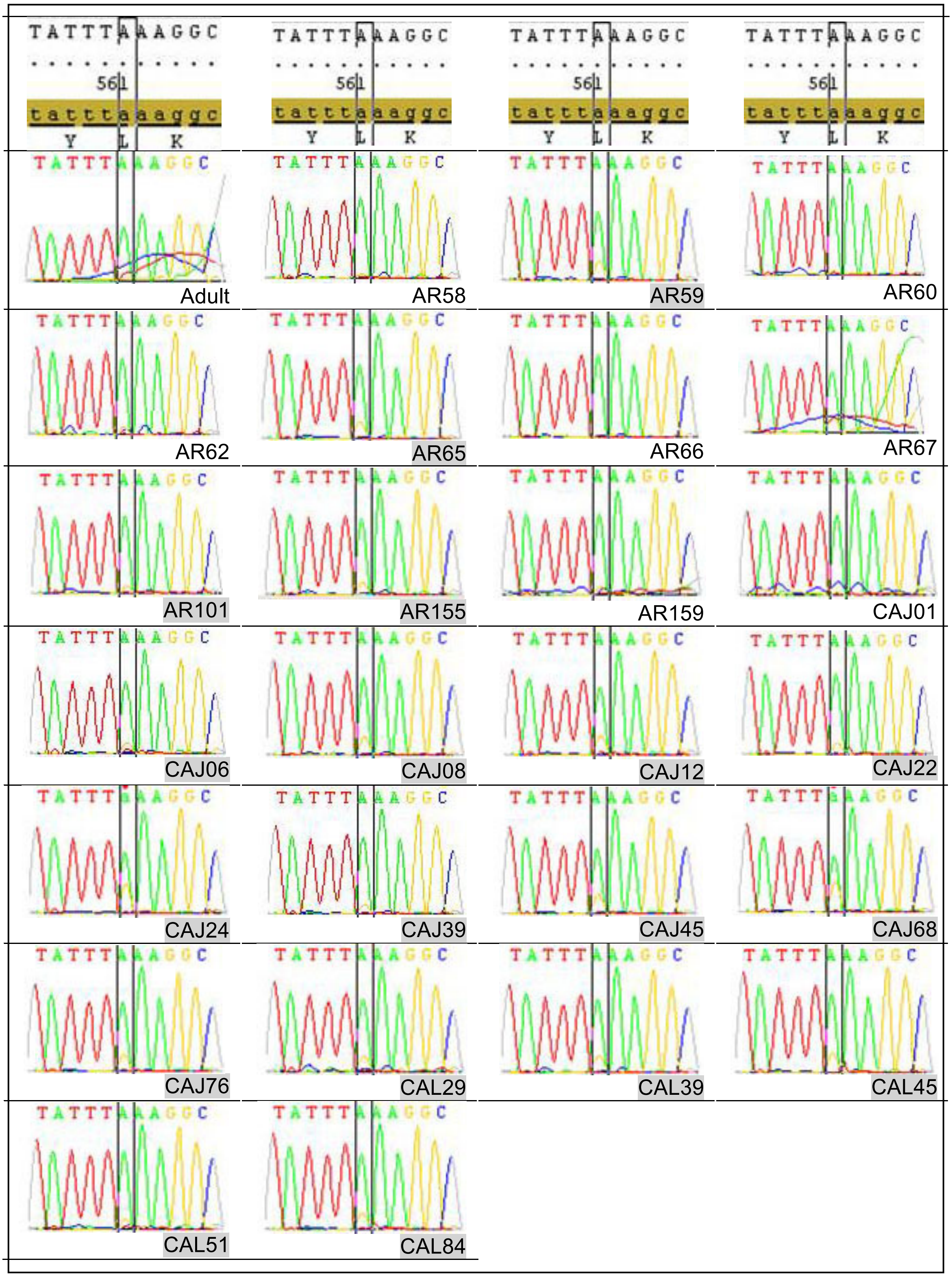

Figure 03: Chromatograms obtained by sequencing the samples of microfilariae pools of *Dirofilaria immitis* sequenced for the β-tubulin gene marking position 561. Samples with double peak highlighted ( ).

Sequencing data

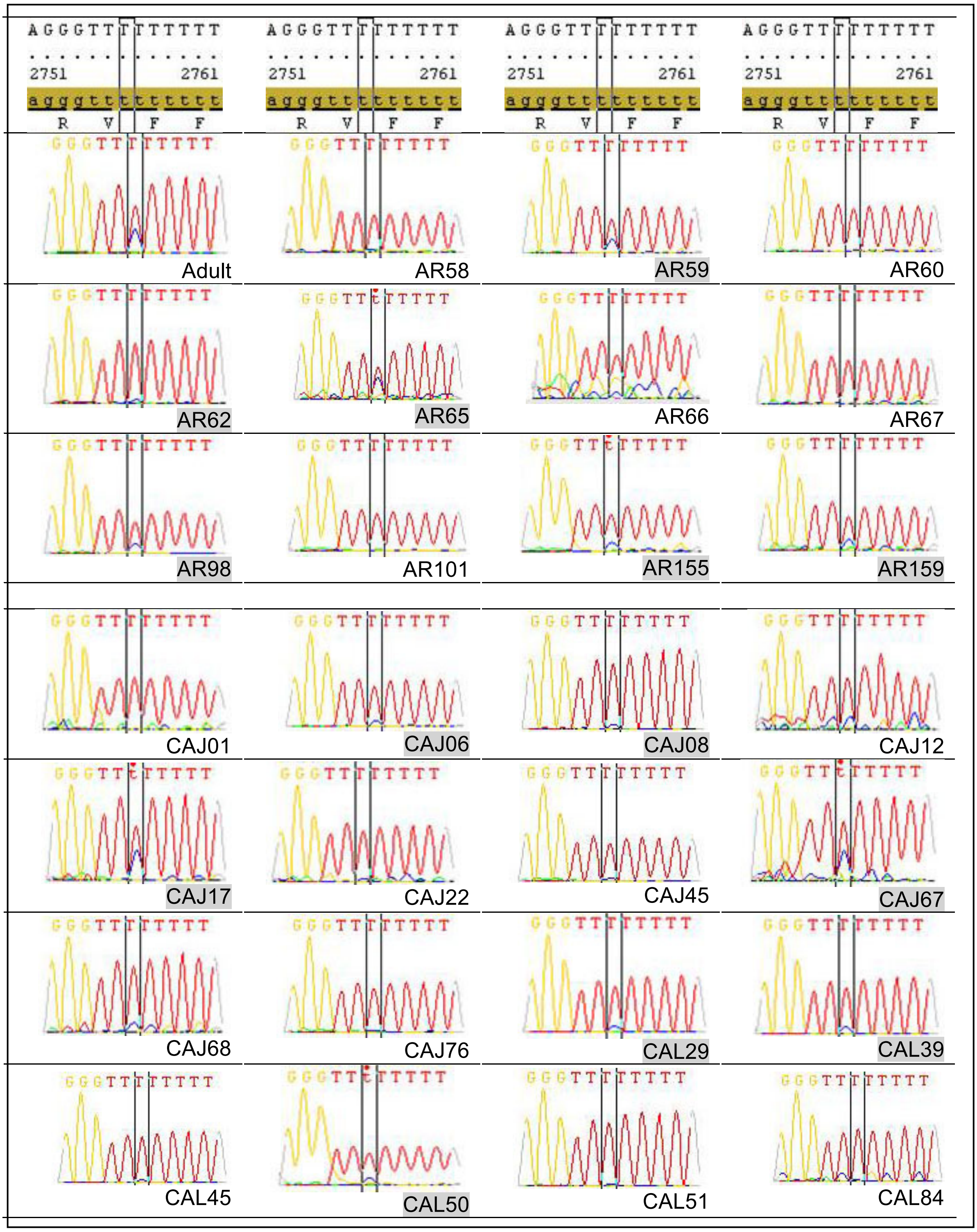

Figure 04: Chromatograms obtained by sequencing the samples of microfilariae pools of *Dirofilaria immitis* sequenced for the β-tubulin gene marking position 2755. Samples with double peak highlighted ( ).
